# N^6^-methyltubercidin gives sterile cure in a cutaneous *Leishmania amazonensis* mouse model

**DOI:** 10.1017/S0031182024000362

**Published:** 2024-04

**Authors:** Cassandra Present, Roberson Donola Girão, Cai Lin, Guy Caljon, Serge Van Calenbergh, Otacilio Moreira, Leonardo Alexandre de Souza Ruivo, Marcos Meuser Batista, Raquel Azevedo, Denise da Gama Jaen Batista, Maria de Nazaré Correia Soeiro

**Affiliations:** 1 Laboratorio de Biologia Celular, Instituto Oswaldo Cruz, Fundação Oswaldo Cruz, Avenida Brasil 4365, Manguinhos, 21040-360 Rio de Janeiro, Brazil; 2 Laboratory of Microbiology, Parasitology and Hygiene (LMPH), University of Antwerp, Universiteitsplein 1, B-2610 Wilrijk, Belgium; 3 Laboratory for Medicinal Chemistry (Campus Heymans), Ghent University, Ottergemsesteenweg 460, B-9000 Gent, Belgium; 4 Laboratório de Virologia e Parasitologia Molecular, Instituto Oswaldo Cruz, Fundação Oswaldo Cruz, Avenida Brasil 4365, Manguinhos, 21040-360 Rio de Janeiro, Brazil

**Keywords:** combination treatment, *in vitro*, *in vivo*, *Leishmania amazonensis*, nucleoside analogues

## Abstract

*Leishmania* is a trypanosomatid parasite that causes skin lesions in its cutaneous form. Current therapies rely on old and expensive drugs, against which the parasites have acquired considerable resistance. Trypanosomatids are unable to synthesize purines relying on salvaging from the host, and nucleoside analogues have emerged as attractive antiparasitic drug candidates. 4-Methyl-7-β-D-ribofuranosyl-7H-pyrrolo[2,3-d]pyrimidine (CL5564), an analogue of tubercidin in which the amine has been replaced by a methyl group, demonstrates activity against *Trypanosoma cruzi* and *Leishmania infantum*. Herein, we investigated its *in vitro* and *in vivo* activity against *L. amazonensis*. CL5564 was 6.5-fold (*P* = 0.0002) more potent than milteforan™ (ML) against intracellular forms in peritoneal mouse macrophages, and highly selective, while combination with ML gave an additive effect. These results stimulated us to study the activity of CL5564 in mouse model of cutaneous Leishmania infection. BALB/c female and male mice infected by *L. amazonensis* treated with CL5564 (10 mg kg^−1^, intralesional route for five days) presented a >93% reduction of paw lesion size likely ML given orally at 40 mg kg^−1^, while the combination (10 + 40 mg kg^−1^ of CL5564 and ML, respectively) caused >96% reduction. The qPCR confirmed the suppression of parasite load, but only the combination approach reached 66% of parasitological cure. These results support additional studies with nucleoside derivatives.

## Graphical abstract

**Figure:**
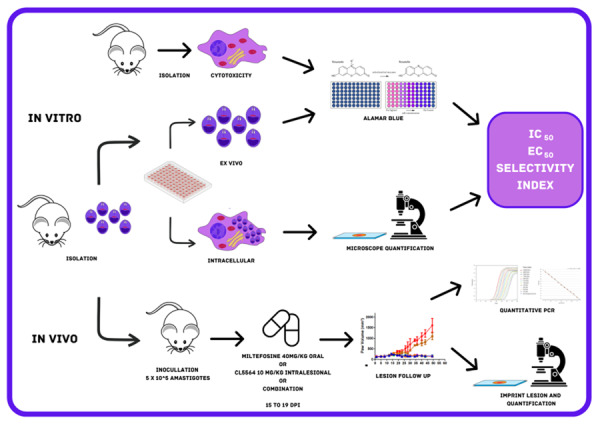

## Introduction

Leishmaniasis is a neglected tropical disease caused by protozoan parasites of the *Leishmania* genus. Various *Leishmania* species have been identified, of which at least 20 can infect humans, whereas other species are not pathogenic to humans. Infection occurs when an infected female sandfly bites to take a blood meal, making leishmaniasis a vector-borne disease (WHO, 2023).

Leishmaniasis is endemic in more than 90 countries, primarily in tropical and subtropical regions but new cases have already been identified in other areas, demonstrating further disease globalization. The WHO estimates that the worldwide prevalence is approximately 12 million, with about 60 000 deaths annually (WHO, 2023).

There are 3 types of disease forms of leishmaniasis: visceral leishmaniasis (VL), cutaneous leishmaniasis (CL) and mucocutaneous leishmaniasis (MCL) and the incubation period varies between 10 days to several months (CDC, 2020). The clinical manifestation depends on several factors such as the *Leishmania* species, vectors and host genetics, co-infections, comorbidities, nutritional stress, environment and climate change (Akbari *et al*., 2021).

*L. amazonensis* is a New World *Leishmania* species belonging to the *L. mexicana* complex (*L. mexicana*, *L. amazonensis*, *L. pifanoi*, *L. garnhami* and *L. venezuelensis*), mainly transmitted by sandflies of the subgenera *Lutzomyia*. It causes CL but is it also an important causative agent of MCL (Akhoundi *et al*., 2016). CL is characterized by skin lesions such as ulcers and sores, which can lead to scars on the face or other exposed areas, leaving disfiguring, life-long scars that bring severe social stigma, particularly for women and children (DNDi, 2020). These lesions can be painful and are often associated with swollen glands (Steverding, 2017). There are approximately 600 000 to 1 million new CL cases each year, making it the most common form of leishmaniasis. MCL causes severe lesions in the mucosae and the mucous membranes of the nose, mouth and throat. It can lead to the destruction of the nasal septum, the lips and the palate, making it the most debilitating disease form (WHO, 2023).

The mechanism of action (MOA) of the current drugs available for the treatment of *Leishmania* infections is not fully understood yet. Moreover, many of them require parenteral administration, and medical monitoring in specialized centres due to common severe side effects. Another concern is the increasing incidence of drug resistance. Thus, the lack of adequate antileishmanial drugs as well as the fast resistance development against the existing drugs, reinforces the urgent development of safer and more effective therapies (de Koning, 2017; CDC, 2020). The low number of drug candidates in the pipeline for leishmaniasis is at least partly related to the lack of interest by pharmaceutical industries due to the low return on investment, as leishmaniasis mainly affects poor populations of low- and middle-income countries (de Vries and Schallig, 2022). Although CL may be self-resolving, this might take several years and will lead to the formation of scar tissue as well as the occurrence of secondary bacterial and/or fungal infections. Moreover, untreated CL might evolve MCL. Conventional treatment of CL includes liposomal amphotericin B, as well as amphotericin B deoxycholate (CDC, 2020). The older pentavalent antimonials and pentamidine remain important in many parts of the world. In addition to amphotericin B, paromomycin and miltefosine (ML) are currently used (CDC, 2020). ML, the only leishmanicidal drug for oral administration, is teratogenic, and presents undesirable side effects such decreased appetite, diarrhoea, fever, nausea, among others.

*Leishmania* parasites multiply fast and require large amounts of purines for the synthesis of nucleic acids (Bouton *et al*., 2021). However, like other protozoan parasites, *Leishmania* is unable to synthesize purines *de novo*, consequently it relies completely on salvaging host purines. This process involves specific transporters and purine salvage enzymes, and strongly differs from the situation in the host cells. Therefore, the transporters and enzymes involved in purine salvage provide interesting targets for the discovery of new antiparasitic agents. Purine (nucleoside) analogues, which inhibit these targets or act as subversive substrates of the salvage enzymes, therefore represent attractive chemotherapeutic candidates (Campagnaro and De Koning, 2020). Several nucleoside analogues have been screened *in vitro* and *in vivo* against trypanosomatid parasites including those causing leishmaniasis, Chagas disease (*Trypanosoma cruzi*) and Sleeping Sickness (*Trypanosoma brucei*) (Mabille *et al*., 2022).

**Figure 1.**
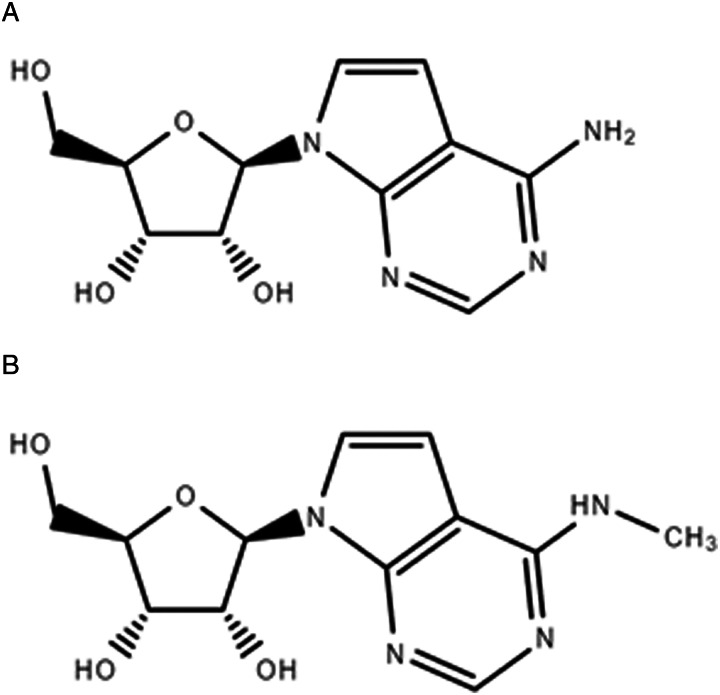
Tubercidin (7-deazapurine) in (A) and 7-deaza-6-methylaminopurine (CL5564) in (B). Adapted from (Lin *et al*., 2022).

Tubercidin (Fig. 1A), a natural adenosine analogue, has trypanocidal effects but is severely toxic to host cells (Aoki *et al*., 2009). Previous research showed that introduction of selected substituents at position 7 improved the activity and selectivity against *T. brucei* but not against *Leishmania* (Hulpia *et al*., 2019). However, monomethyl substitution of the exocyclic amine of tubercidin affords an analogue that retains potent and selective anti-*Trypanosoma cruzi* and anti-leishmanial activity (*L. infantum*), but with a favourable selectivity profile as opposed to tubercidin. Although this analogue (named CL5564, Fig. 1B) did not show obvious *in vitro* cytotoxicity, it caused severe toxicity when assayed in an acute hamster model in an oral dosing of 50 mg kg^−1^ s.i.d (Lin *et al*., 2022).

In this study, the leishmanicidal activity of CL5564 was further assessed against *L. amazonensis in vitro* and *in vivo*. The compound proved to display potent *in vitro* activity, and in combination with ML, CL5564 gave parasitological cure in 66% of mice with a *L. amazonensis* cutaneous infection after only 5 days of oral and intralesional drug administration, respectively.

## Materials and methods

### Animals

Mice (male and female BALB/c and Swiss Webster) were obtained from the animal facilities of ICTB (Institute of Science and Biomodels Technology, Fiocruz, Rio de Janeiro, Brazil), housed up to 5 per cage, kept in a room at 20–24°C under a 12 h light and 12 h dark cycle, and provided with sterile water and chow *ad libitum*. The animals were acclimated for 7 days before performing the assays. All procedures were done following Biosafety Guidelines in compliance with the Fiocruz and all animal procedures approved by the Committee of Ethics for the Use of Animals (CEUA numbers L038-2017, L-038/2017-A4).

### Parasites

Male BALB/c mice were inoculated in the foot paws (subcutaneously) with 20 μL containing 10^6^ amastigotes of *Leishmania* (L.) *amazonensis* (MHOM/BR/77/LTB0016). Thirty days post-infection, the skin lesions were removed aseptically, and mechanically dispersed by pipetting and the amastigotes purified as reported (Santos *et al*., 2022).

### Peritoneal mouse macrophage (PMM)

PMM were obtained from Swiss male mice (18–20 g) previously stimulated with 3% thioglycolate as reported (Santos *et al*., 2022). Four days after stimulation, PMM were collected by rinsing the mice's peritoneum with 10 mL of RPMI 1640 with phenol red, the cells seeded in 24- (3 × 10^5^ cells well^−1^) and 96- (5 × 10^4^ cells well^−1^) well plates and maintained for infection and cytotoxicity assays, respectively. PMM cell cultures were sustained in a 37°C incubator with 5% CO_2_ in RPMI 1640 medium (pH 7.2–7.4) with phenol red and without phenol red (Gibco BRL) for infection analysis and cytotoxicity assays, respectively, supplemented with 1% (v/v) L-glutamine 200 mm, 1% (v/v) penicillin–streptomycin (10 000 U mL^−1^ to 10 mg mL^−1^) and 10% (v/v) FBS (Santos *et al*., 2022).

### Compounds

CL5564 was synthesized as reported (Lin *et al*., 2022). A stock solution (50 mm) of CL5564 was prepared in DMSO, while miltefosine (Sigma–Aldrich, #M5571) was prepared in ultrapure and sterile water (Santos *et al*., 2022). The concentration of DMSO was lower than 0.6% for all *in vitro* experiments to prevent non-specific toxicity to the host cells (Romanha *et al*., 2010). For *in vivo,* CL5564 was prepared in 10% ethanol in citrate buffer 0.1 M pH 3.02 and Milteforan™ (Virbac) diluted in ultrapure and sterile water as reported (Lin *et al*., 2022).

### Cytotoxicity assay

Toxicity towards PMM was analysed by adding a 1:2 dilution of each compound (400–3.13 μm, 8 concentrations) into uninfected cultures and incubating for 48 and 120 h at 37°C. The cellular viability was evaluated using PrestoBlue™ (560–590 nm) (Santos *et al*., 2022). The percentage of reduction in the host cell viability was calculated in a non-linear regression curve to calculate the CC_50_ value (minimum concentration that reduces 50% cellular viability).

### Activity against free amastigotes (ex vivo assay)

The *ex vivo* assays were conducted to evaluate the direct effect of both compounds on the free amastigotes purified directly form mice lesions as reported (Santos *et al*., 2022). 1 × 10^6^ parasites per well (100 μL) were seeded in a 96-well plate and then 100 μL of each compound was added at the double final concentration (up to 40 μm). After 48 and 120 h of incubation at 32°C, the parasite viability was evaluated using light microscopy and by PrestoBlue™ reaction (560–590 nm) to calculate the IC_50_ value by a non-linear regression curve (Santos *et al*., 2022).

### Activity against intracellular amastigotes in PMM

Infection analyses were conducted to investigate the *in vitro* activity against intracellular amastigotes of *L. amazonensis*. PMM cultures (3 × 10^5^ cells well^−1^) were infected for 2 h with purified amastigotes (5:1 parasites:cell ratio) at 37°C, and then rinsed twice to remove non-internalized parasites. Next, a 1:3 dilution of each compound (up to 30 μm) was added and further incubated at 37°C. After 48 h, the cultures were washed twice with phosphate buffered saline (PBS), fixed with Bouin for 10 min., and stained with Giemsa (1:10 dilution) for 40 min. (Sigma–Aldrich, 32884) (Araújo-Jorge and Castro, 2000; Santos *et al*., 2022). Light microscopy quantification was performed by counting (300 cells per well) the percentage of infected PMM and the number of parasites per infected PMM to calculate the infection index (percentage of infected PMM multiplied by the average number of intracellular parasites per infected PMM). The IC_50_ and IC_90_ values (minimum concentration able to reduce the parasite load with 50% and 90%) were determined based on the infection indexes as reported (Santos *et al*., 2022).

### In vitro combination assays

*In vitro* drug interactions of ML and CL5564 were performed using *ex vivo L. amazonensis* amastigotes (Fivelman *et al*., 2004; Santos *et al*., 2022). Fractional inhibitory concentration indexes (FICI) and the sum of FICIs (∑FICI) were calculated as follows: FICI values = IC_50_ of the combo/IC_50_ of each compound alone. An overall ∑FICI was then determined and used to classify the nature of each interaction: ∑FICI ⩽ 0.5 = synergism; 0.5 < ∑FICI ⩽ 4.0 = additive (no interaction); ∑FICI > 4.0 = antagonism (Odds, 2003; Rocha-Hasler *et al.,* 2021).

### In vivo activity studies

BALB/c mice were infected in the back right paw region with 5 × 10^5^ amastigotes of *L. amazonensis* purified from animal lesions. Two males and two females were used in vehicle treated and Milteforan™ group each, while for CL5564 and combination groups, 3 males and 3 females were used. The size of the paw lesion was measured every 2 days using a paquimeter (Santos *et al*., 2020). From 15 to 19 days post-infection (dpi), the animals were treated once a day with milteforan™ 40 mg kg^−1^ orally by gavage, and with CL5564 at 10 mg kg^−1^ administrated intralesionally. Also, a combo using milteforan™ 40 mg kg^−1^ plus CL5564 10 mg kg^−1^ was administrated by gavage and intralesionally, respectively. For control, mice were treated intralesionally with the vehicle consisting of 10% ethanol in citrate buffer 0.1 M pH 3.02. The animals were monitored until 50 dpi and then euthanized. The paws' lesions were then collected for *imprint* analysis performed by Giemsa staining and light microscopy evaluation of the parasite number in 10 random fields at 100× magnification (Santos *et al*., 2022). Also, the lesion samples were processed for qPCR quantification to determine the total parasite load. Briefly, after homogenization of the skin lesion sample using the Tissueruptor II (Qiagen, Hilden, Germany) for 30 s in 200 μL of tissue lysis buffer (Roche, Basel, Switzerland), DNA was isolated using the High Pure PCR Template Preparation kit (Roche, Basel, Switzerland) according to manufacturer's instructions. At the final step of the protocol, DNA was eluted in 100 μL and stored at −20°C until use. The real-time PCR assays were performed using TaqMan system with primers and probe designed for the 18S rDNA target in *Leishmania* (Filgueira *et al*., 2020). In parallel, a TaqMan assay for the mouse GAPDH gene was used as the internal control and to normalize the parasitic load [Mouse GAPD (GAPDH) Endogenous Control – VIC/MGB probe, primer limited. Cat. No: 4352339E (Applied Biosystems, Foster City, USA)]. Reactions were carried out using 10 μL FastStart Universal Master Mix [2X] (Roche, Basel, Switzerland), 150 nm primer 18S rDNA F, 300 nm primer 18S rDNA R, 200 nm 18S rDNA Tq (FAM/NFQ-MGB) and 5 μL DNA, in a final volume of 20 μL. In parallel, assays containing 10 μL FastStart Universal Master Mix [2X] (Roche, Basel, Switzerland), 2 μL mouse GAPD (GAPDH) endogenous control [20X] (VIC/NFQ/MGB) and 5 μL DNA, in a final volume of 20 μL were done. Cycling conditions were a first step at 95°C for 5 min, followed by 40 cycles at 94°C for 15 s and at 60°C for 1 min. The amplifications were carried out in a Quantstudio 3 Real Time PCR system (Applied Biosystems, Foster City, California, USA) and the threshold was set at 0.02 for all targets. For the absolute quantification by real-time qPCR, mouse skin samples (30 mg) were spiked with 10^6^ *L. amazonensis* (MHOM/BR/77/LTB0016) promastigotes before DNA extraction. Standard curves were prepared by DNA serial dilutions in a 1:10 dilution factor, and used in each qPCR plate, reaching from 10^5^ to 1 parasite equivalents/reaction and from 50 to 5 × 10^−3^ mg of mouse tissue. The parasitic load was normalized by the mice tissue load, being expressed as equivalents of parasite/mg of mouse tissue.

Negative and positive controls were used in all experiments. In each batch of DNA extraction (up to 12 samples), one tube containing 200 μL molecular biology water, instead of sample, was used as negative control. In addition, in each real-time PCR plate, 2 wells containing 5 μL of ultrapure water instead the DNA sample were used as negative template control (NTC). As positive controls, 5 μL each of *Leishmania braziliensis* DNA (at 1 pg μL^−1^ and 100 fg μL^−1^) were used in each real-time PCR plate. Each sample was assayed in technical duplicates. A sample was considered positive (detectable Leishmania DNA) when the amplification curve for the *Leishmania* target exceeded the fluorescence threshold (set at 0.02) during the 40 PCR cycles, resulting in a Ct value. A sample was considered negative (non-detectable *Leishmania* DNA) when the amplification curve did not exceed the fluorescence threshold, resulting in Ct absence during the 40 cycles.

### Statistical analysis

Statistical analysis was performed using a unifactorial ANOVA analysis with a significance level of *P*  ⩽ 0.05. Three independent assays (in triplicates) were performed for the *ex vivo* and cytotoxicity approaches. For the *in vitro* intracellular amastigote assays, 2 independent experiments were performed in duplicates. For *in vivo,* a proof of concept was performed using *n* = 3 (females and male mice) for the CL5564 and CL + milteforan groups while *n* = 2 was included for vehicle and milteforan-treated mice.

## Results

### Cytotoxicity analysis

First, the toxicity profile of miltefosine (ML) and CL5564 was investigated towards PMM, and both gave a dose-dependent toxic effect. The reference drug displayed a CC_50_ value of 139 μm while CL5564 showed a CC_50_ value of 155 μm after 48 h of incubation (Table 1). Since the cytotoxicity assays showed a toxicity profile of the purine analogue towards the PMM after 120 h of incubation, the drug exposure in the following assays was restricted to 48 h.

**Table 1.** Leishmanicidal activity on *ex vivo* and intracellular amastigotes of *Leishmania amazonensis* and toxicity on peritoneal mouse macrophages exposed for 48 h to the studied drugs

	IC_50_ (μm)		IC_90_ (μm)	Toxicity	
	*Ex vivo*	Intracellular	Intracellular	CC_50_ (μm)	SI
Miltefosine	1.54 ± 0.86	3.20 ± 0.66	10.49 ± 3.87	138.87 ± 15.70	43
CL5564	0.41 ± 0.41^a^	0.56 ± 0^b^	1.01 ± 0^c^	155.40 ± 75.39^d^	278

The selectivity index (SI) is relative to intracellular IC_50_ and CC_50_ on macrophages.

*Note*: Statistical analysis comparing the activity of CL5564 with Miltefosine in each different experimental conditions (*ex vivo*, intracellular, and toxicity in PMM): ^a^*P* = 0.03, ^b^*P* = 0.0002, ^c^*P* = 0.003 and ^d^*P* = 0.002

### Activity against intracellular amastigotes in PMM

The activity of the compounds was inspected against the intracellular forms of *L. amazonensis* in PMM. Our findings demonstrated that infected and untreated cultures reached 91% of parasitized host cells with about 6 intracellular parasites per infected cell, resulting in an infection index of 588. When ML was added, a dose-dependent reduction in the percentage of infected PMM was observed, but not in the number of parasites per infected host cell, except for the concentrations ⩾10 μm. On the other hand, CL5564 resulted in a higher pronounced dose-dependent reduction in both the percentage of infected cells and the number of parasites per infected host cell, confirming its high leishmanicidal activity (Table 1).

With an IC_50_-value of 0.56 μm CL5564 was significantly (*P* = 0.0002) more active than ML IC_50_ = 3.2 μm). The IC_90_ value of CL5564 (1.01 μm) was 10-fold lower (*P* = 0.003) than that of ML (10.49 μm), as well as its selectivity index (278 *vs* 43 for ML), further indicating its potential as a leishmanicidal drug candidate (Table 1).

### Activity against free amastigotes (ex vivo assay)

Next, assays were performed using *ex vivo* amastigotes obtained directly from mice lesions and incubated at 32°C for 48 h with different concentrations of ML and CL5564 under well-standardized protocols (Santos *et al*., 2022). As depicted in Table 1, our findings showed low IC_50_ values for all studied conditions: CL5564 gave an IC_50_ of 0.41 μm, being significantly more potent (3.8-fold) than ML (IC_50_ value of 1.54 μm) (*P* = 0.03).

### In vitro combination assays

**Figure 2.**
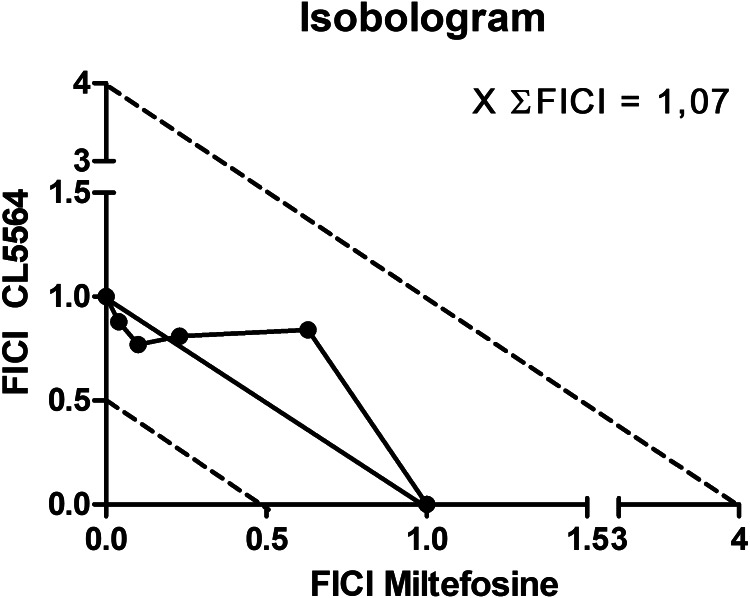
Isobologram of miltefosine and CL5564 used in combination. Result shows an additive effect.

The combination of CL5564 plus ML was performed using free *ex vivo* amastigotes. The results showed an overall additive effect with the mean of the sum FICI equal to 1.07 after 48 h of incubation (Fig. 2).

### In vivo combination assays

**Figure 3.**
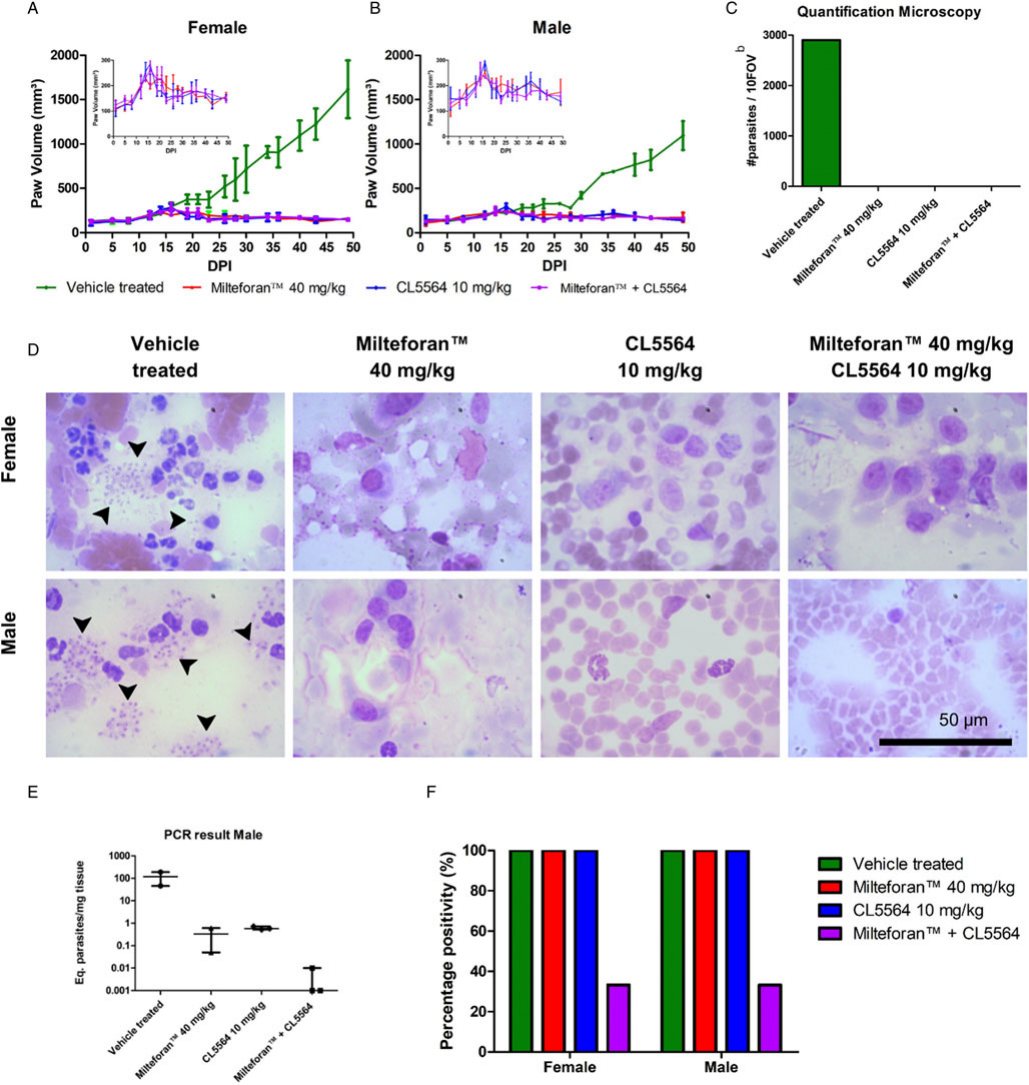
CL5564 and milteforan^TM^ effect, alone or in combo, using a mouse cutaneous leishmaniasis model of *L. amazonensis* infection. In (A) and (B), the growth of lesion volume monitored with a paquimeter for female and male mice, respectively. The inset graph is a zoom of the drug-treated groups. In (C) is the number of amastigotes' quantification by light microscopy in 10 FOV; (D) representative images of imprint paws from the different animal groups; and (E) represents the determination of parasite load by qPCR and (F) the qPCR positivity percentage in all groups.

Lastly, a proof of concept was performed using *L. amazonensis* infection in BALB/c female and male mice using intralesional administration of CL5564 (10 mg kg day^−1^), which corresponds to a p.o. dosing regimen that was previously shown to be well-tolerated (Lin *et al*., 2022). The animal lesions were followed up for 50 dpi that corresponded to the endpoint. In vehicle-treated groups, the lesion increased significantly after 15 dpi, being larger in female than male mice (Fig. 3A and B). The intralesional administration of CL5564 at 10 mg kg^−1^ gave a similar efficacy as milteforan™, as the lesion totally regressed (Fig. 3A and B) in both genders. The reduction in the lesion size was 93–96% and 97–100% for milteforan™ and CL5564, respectively. Also, a combo of ML and CL5564 afforded almost complete lesion reduction reaching >97% (Fig. 3A and B). The analysis by light microscopy of the imprints confirmed the parasite load suppression in the lesions: while the number of amastigotes in vehicle-treated cultures was about 290 amastigotes/field, no parasites could be found in the cultures of the treated animals, even after exploring more than 50 random fields, regardless of the studied gender and the compound given alone or in combo (Fig. 3C and D). To further investigate parasitological cure of the treated mice, a quantitative PCR was performed. The leishmania load in male mice reached a median of 117.9 Eq. of parasite/mg tissue in the vehicle-treated group, while milteforan™, CL5564 and combo gave 0.245, 0.403 and 0.002 Eq. parasite/mg tissue, respectively (Fig. 3E). In males, we observed a 357 and 197-fold reduction in the parasite load in the groups treated with milteforan and CL5564, respectively, when compared to vehicle-treated control groups. The combo showed a higher reduction in the parasite load, being 45 800-fold lower than vehicle-treated males, which proved 165- and 300-fold better than with milteforan™ and CL alone, respectively (Fig. 3E). In females, we also observed reductions (80 and 100-fold) in the parasite load of the combo as compared to the compounds alone (data not shown). In the combos of both groups (females and males), 2/3 of the animals had negative qPCR, denoting sterile cure. It is important to highlight that in all animals treated with the nucleoside derivative, no side effects were noticed.

## Discussion

According to WHO, leishmaniasis is a neglected tropical disease (NTD) with a broad spectrum of clinical manifestations such as cutaneous, visceral and mucocutaneous presentations caused by more than 20 different species of the genus *Leishmania* that can be transmitted by over 90 sandfly species (WHO, 2023).

The disease development is dependent on several factors such as malnutrition, poor housing and deficient sanitary conditions, host and parasite genetics, environmental and climate changes, co-infections, among others. In fact, there is a straight correlation between poverty and NTDs, keeping alive the poverty-disease cycle (Teixeira-Neto *et al*., 2014; de Angeli Dutra *et al*., 2022; de Vries and Schallig, 2022).

Due to the limited access and available medical care units, the diagnosis is frequently performed when the disease has already progressed, reducing the chances of a successful therapy. If left untreated, the most aggressive form VL may be fatal. Still, the other 2 clinical tegument forms also represent serious challengers since they result in ulcers, scars, disabilities, stigmas and are frequently associated with secondary infections that amplify the disease condition and delay the patient's recovery (CDC, 2020).

Other important issues are related to the need to implemented and sustained governmental and Mundial politics to strengthen surveillance and control of parasite transmission, as well as the urgent need for new therapeutic options (Baneth *et al*., 2020).

Available treatments for *in situ* (topic and intralesional) and systemic administration have limited efficacy and are mainly composed of old and toxic drugs. Moreover, these drugs also cause side effects, are costly, and have become ineffective against increasing drug-resistant parasite populations (de Koning, 2017; Garza-Tovar *et al*., 2020). Thus, novel therapeutic options are necessary, and phenotypic screening is believed to be the best option to identify novel hits and lead compounds.

Kinetoplastid parasites such as *T. brucei*, *T. cruzi* and *Leishmania* sp. are auxotrophic for purines, relying on the uptake and assimilation of host purines. This makes the screening of purine nucleoside analogues as chemotherapeutic agents against such protozoal infections very rewarding (el Kouni, 2003).

This study further explored the potential of CL5564, a methylated analogue of tubercidin, previously found to exhibit promising *in vitro* activity and selectivity against both *T. cruzi* and *L. infantum*. This analogue was already explored in an acute Chagas disease mouse model. Although it dose-dependently reduced parasitemia peak, at the highest dose (50 mg kg day^−1^), it failed to protect against mice mortality, possibly due to acute drug toxicity (Lin *et al*., 2022). Also, we also noticed acute toxicity aspects (such as loss of animal weight) in a hamster model of visceral leishmaniasis that received oral administration of CL5564 at 50 mg kg day^−1^. However, in the mouse model of *T. cruzi* acute infection, the lower doses (such as 12.5 and 25 mg kg day^−1^) did not induce noticeable side effects. Thus, besides being well-tolerated (12.5 mg kg day^−1^), CL5564 sustained its efficacy, being able to reduce *T. cruzi* parasite load by 75% and providing more than 83% of animal survival, while vehicle-treated group displayed 100% mice mortality (Lin *et al*., 2022). Then, presently CL5564 was given at non-toxic dose (10 mg kg day^−1^) by intralesional route directly into the mice lesion as this route has already been under clinical use for some leishmanicidal drugs such as pentavalent antimonial. Under this protocol, presently no animal side effect was observed.

The main objective of this study was to further investigate the leishmanicidal activity of the 7-deaza-6-methylaminopurine (CL5564) alone or in combination with the reference drug miltefosine using *L. amazonensis* parasites.

The cytotoxicity of CL5564 was assessed using PMM host cells obtained from mice stimulated with thioglycolate. Miltefosine (ML) and CL5564 displayed similar CC_50_ values of 139 and 155 μm after 48 h of treatment, respectively, which is in line previously reported data (Santos *et al*., 2020, 2022; Lin *et al*., 2022).

CL5564 proved more potent than ML against intracellular (PMM) *L. amazonensis* infection (IC_50_ values of 0.56 and 3.20 μm after 48 h of incubation, respectively). Notably, this translated into a superior selectivity index for CL5564 (278 *vs* 43). These results met the criteria for further exploration of the efficacy of CL5564 *in vivo* (Strovel *et al*., 2004; GHITF, 2023).

The earlier observed toxic events when administered orally at 50 mg kg^−1^ (Lin *et al*., 2022), led us to assess the effect of a relatively low dose (10 mg kg day^−1^) of CL5564 upon intralesional administration (Soto *et al*., 2018). In fact, *in situ* administration of leishmanicidal and immunomodulatory agents has been recommended by the WHO and by the Pan American Health Organization (PAHO) as an alternative to the routine systemic administration in patients with at least 5 lesions smaller than 4 cm in diameter, especially when the face or joints are not affected. This alternative administration is easy and cheap and poses fewer risks of toxic events. The *in situ* administration involves protocols employing thermotherapy, cryotherapy, and may be particularly relevant for localized clinical forms of the disease (Garza-Tovar *et al*., 2020; Azim *et al*., 2021; Madusanka *et al*., 2022).

Another promising approach is the drug combination that has been used for several diseases including those of infection origin. Combos may act on different and/or complementary targets, allowing the use of lower drug concentrations and thus reducing toxic events (Wiwanitkit, 2012; Hendrickx *et al*., 2017; Gaibani *et al*., 2019; Ianevski *et al*., 2021; Mao *et al*., 2021).

Besides targeting distinct and/or complementary mechanisms of action, which minimizes the chances of parasite drug resistance, drug combination may reach higher efficacy and lower incidence of adverse and side effects. In addition, lower doses also reduce the costs associated with research and development of new entities or separate production processes (Ferraz *et al*., 2022). In fact, different combos have been successfully reported in clinical trials (Azim *et al*., 2021) as well as in experimental models such as the milteforan^TM^ plus paromomycin in *L. infantum* infection (Hendrickx *et al*., 2017).

However, due to the limitation of evaluating many drug concentrations using infected PMM, *ex vivo* amastigotes purified from mice lesions were chosen for the initial evaluation of drug combination using the fixed-ratio method under well-standardized protocols (Santos *et al*., 2020).

CL5564 demonstrated an almost 4-fold lower IC_50_ value against the *ex vivo* amastigotes than ML. Upon combination an additive effect was observed.

In BALB/c mice inoculated with the same *Leishmania* strain, 10 mg kg^−1^ of CL5564 gave a similar efficacy as 40 mg kg^−1^ milteforan^TM^, with a reduction higher than 93% after only 5 days of intralesional administration. Quantification of the amastigotes in animal lesions through light microscopy of the *imprints* collected from the mice paws confirmed the control of parasite burden in all treated groups. However, quantitative PCR indicated that the combo was about 128-fold more effective than the separate drugs, resulting in 66% sterile cure, regardless of the animal gender. The remaining positive animals (34%) of the combo group presented a very low parasite burden, close to the limit of detection.

Developing safer and shorter treatments given orally is the ideal goal for treating any neglected tropical disease (Katsuno *et al*., 2015). However, as stated by DNDi, while not achieving an ideal oral drug, ‘few doses by parenteral route’ is acceptable as target product profile for CL.

Our present findings justify further studies with nucleoside analogues alone or in combination with reference drugs for CL, also exploring parasitism reactivation as well as the host immunological profile. In fact, research studies performed by different methodologies represent an academic cornerstone in the proposal of novel therapies for NTD that receive little financial support for the development of new drug despite affecting more than one billion people worldwide, especially those more vulnerable (women and children), which urgently need faster, safer, and more effective therapies.

## Data Availability

The data are available at Cellular Biology Laboratory/IOC/Fiocruz.
